# Metagenomic next-generation sequencing indicates more precise pathogens in patients with pulmonary infection: A retrospective study

**DOI:** 10.3389/fcimb.2022.977591

**Published:** 2022-10-07

**Authors:** Dengfeng Wu, Wei Wang, Qiufen Xun, Hongluan Wang, Jiarong Liu, Ziqing Zhong, Chao Ouyang, Qing Yang

**Affiliations:** ^1^ Department of Respiratory and Critical Care Medicine, The Second Affiliated Hospital of Nanchang University, Nanchang, China; ^2^ Department of Respiratory and Critical Care Medicine, Jiangxi Provincial People’s Hospital, Nanchang, China; ^3^ Department of Nephrology Medicine, The Second Affiliated Hospital of Nanchang University, Nanchang, China

**Keywords:** metagenomic next-generation sequencing, bronchoalveolar lavage fluid, pulmonary infection, etiology diagnosis, comorbidities

## Abstract

**Background:**

Timely identification of causative pathogens is important for the diagnosis and treatment of pulmonary infections. Metagenomic next-generation sequencing (mNGS), a novel approach to pathogen detection, can directly sequence nucleic acids of specimens, providing a wide range of microbial profile. The purpose of this study was to evaluate the diagnostic performance of mNGS in the bronchoalveolar lavage fluid (BALF) of patients with suspected pulmonary infection.

**Methods:**

From April 2019 to September 2021, 502 patients with suspected pneumonia, who underwent both mNGS of BALF and conventional microbiological tests (CMTs), were classified into different groups based on comorbidities. The diagnostic performances of mNGS and CMTs were compared. Comprehensive clinical analysis was used as the reference standard.

**Results:**

The diagnostic accuracy and sensitivity of mNGS were 74.9% (95% confidence interval [CI], 71.7-78.7%) and 72.5% (95% CI, 68.2-76.8%) respectively, outperformed those of CMTs (36.9% diagnostic accuracy, 25.4% sensitivity). For most pathogens, the detection rate of mNGS was higher than that of CMTs. Polymicrobial infections most often occurred in immunocompromised patients (22.1%). Only 2.3% patients without underlying diseases developed polymicrobial infections. Additionally, the spectrums of pathogens also varied among the different groups. We found the positive predictive values (PPV) to be dependent upon both the pathogen of interest as well as the immunologic status of the patient (e.g., the PPV of Mycobacterium tuberculosis was 94.9% while the PPV of Pneumocystis jirovecii in immunocompetent individuals was 12.8%). This information can help physicians interpret mNGS results.

**Conclusion:**

mNGS of BALF can greatly enhance the accuracy and detection rate of pathogens in patients with pulmonary infections. Moreover, the comorbidities and types of pathogens should be taken consideration when interpreting the results of mNGS.

## Introduction

Pulmonary infection remains one of the leading causes of morbidity and mortality worldwide (GBD 2016 Causes of Death Collaborators, 2017). Accurate antimicrobial treatment can improve the cure rate, reduce broad-spectrum antibiotic use, and decrease medical costs. Therefore, timely identification of causative pathogens is critical for improving the clinical prognosis (Metlay et al., 2019). Conventional cultures are routinely used to detect the pathogens in pulmonary infections. However, this method is time-consuming and exhibit a low detection rate. Culture-independent techniques (polymerase chain reaction[PCR] and serological testing) have been proven useful for broadening the scope of detectable pathogens and increasing the detection rate; however, they require clinicians to speculate the pathogenic species (Jain et al., 2015, Gadsby et al., 2016).Hence, a rapid and unbiased diagnostic approach is urgently required.

Metagenomic next-generation sequencing (mNGS), a nucleic acid sequencing technique, can detect nearly all pathogens in theory (Chiu and Miller, 2019; Gu et al., 2019). To date, mNGS has been increasingly applied to pulmonary infections and has shown better diagnostic performance than cultures, especially in immunocompromised patients and in patients with severe pneumonia (Dickson et al., 2017, Han et al., 2019, Xie et al., 2019). Optimal lower respiratory tract specimens are crucial for microbial diagnosis. Bronchoalveolar lavage fluid (BALF) is an easily available clinical specimen that can avoid contamination by oropharyngeal flora when compared with sputum, enabling higher sensitivity than blood (Dubourg et al., 2015, Chen et al., 2020). However, there are still numerous challenges when applying mNGS of BALF samples in pulmonary infection, because of the presence of commensal microbes in the respiratory tract and the complexity of pulmonary infections (Dickson et al., 2016, Dickson et al., 2017). Thus, further investigation on the application of mNGS for BALF samples is required for identifying pathogens contributing to pulmonary infections.

In the present study, we evaluated the value of mNGS in the diagnosis of patients suspected with pulmonary infection compared to that of conventional microbiological tests (CMTs). Moreover, we assessed the spectrum of pathogens in patients with different comorbidities. The positive predictive values (PPVs) of different pathogens were calculated to evaluate the reliability of positive results of mNGS.

## Methods

### Study design and population

Adult patients (aged > 18 years) with suspected pulmonary infection from The Second Affiliated Hospital of Nanchang University and Jiangxi Provincial People’s Hospital between April 2019 and September 2021 were retrospectively reviewed. Age, sex, comorbidities, laboratory test results, lung images, antibiotic therapy, and patient outcomes were recorded. Patients suspected with pulmonary infection required new-onset shadows on chest images and at least one of the following symptoms: 1) cough, dyspnea, or other respiratory symptoms; 2) fever; and 3) leukocytosis or leukocytopenia. 502 patients were enrolled in the study. All patients underwent bronchoscopy to obtain BALF samples. Both CMTs and mNGS of BALF were performed to detect pathogens. Based on the comorbidities, 502 patients were classified into four groups: 1) immunocompromised group: patients were viewed as the immunosuppressed host; 2) bronchiectasis group: patients were diagnosed with bronchiectasis (destructive pneumonophthisis patients were also classified into this group because of the structural lesions in the lung); 3) other comorbidities group: patients with other comorbidities which can increase the risk of pulmonary infections (including diabetes, chronic obstructive pulmonary disease, interstitial lung disease, bronchial asthma, cerebrovascular disease, lung cancer treated with targeted therapy or surgery, and liver cirrhosis, apart from the above two groups) (Martino et al., 2005, Wu et al., 2016); and 4) simple pulmonary infection group: patients without previous underlying diseases. Immunosuppressed hosts were required to meet any of the following criteria: 1) long-term steroid therapy (> 20 mg/d prednisone-equivalent and cumulative dose≥600 mg), 2) autoimmune disease treated with immunosuppressive agents or cytotoxic drugs, 3) solid-organ transplantation, 4) recent chemotherapy during the last month, 5) hematological malignancy, 6) agranulocytosis, and 7) human immunodeficiency virus (HIV) infection (Ramirez et al., 2020, Peng et al., 2021). For bronchiectasis patients, computed tomography of the chest had to fulfil at least one of the following criteria: 1) the inner diameter of the bronchus/the diameter of the concomitant pulmonary artery > 1; 2) the bronchi do not become thinner from the center to the periphery; 3) the bronchioles could be seen in the range of 1 cm from the peripheral pleura (Bonavita and Naidich, 2012).

### Conventional microbiological tests

Conventional microbiological tests (CMTs) included a smear and culture of bacteria and fungi, acid-fast stain, PCR, Grocott’s methenamine silver stain, and *Cryptococcus* capsular polysaccharide test. Other methods, such as galactomannan antigen and (1,3)-β-D-glucans test for fungi, tuberculin skin test, and enzyme-linked immune spot for *Mycobacterium tuberculosis*, were not regarded as etiologically confirmed methods.

### Clinical comprehensive analysis was regarded as the reference standard

Two experienced clinicians in the management of pulmonary infection independently reviewed the medical records of all patients along with the results of mNGS. First, clinicians judged whether the patients had infectious or non-infectious diseases. Second, the causative pathogens were determined by a comprehensive analysis based on clinical manifestations, test results, chest radiology, and treatment response. The determination of the causative pathogens should at least meet the following criteria: etiological examination (including conventional microbiological tests and mNGS) detected the pathogens. Characteristic medical imaging and clinical manifestations can be helpful in the diagnosis of causative pathogens.

### Sample processing

Once acquired, 2mL BALF was placed in a ribozyme free centrifuge tube at -20°C and sent to BGI-Hua da (Wuhan, China) on dry ice for sequencing and bioinformatics analysis on the same day. A1.5mL microcentrifuge tube with 0.6mL BALF and 250μL 0.5mm glass bead were attached to a horizontal platform on a vortex mixer and agitated vigorously at 2800-3200 rpm for 30 min. 7.2μL lysozyme was then added for wall-breaking reaction. DNA was extracted using a TIANamp Micro DNA Kit (DP316, TIANGEN BIOTECH) according to the manufacturer’s recommendations.

The remaining BALF was sent to a clinical microbiology laboratory. Bacterial cultures, fungal cultures, acid-fast bacteria (AFB) staining, PCRs of mycobacteria and gram staining were performed for every samples. Bacterial cultures were set up on MacConkey and blood agar plates. Fungal cultures were set up on liquid Sabouraud medium. AFB staining was performed using the Ziehl-Neelsen staining method. PCRs for virus, bacteria, and fungal were performed according to the clinician’s discretion.

### Sequencing and bioinformatic analysis

The extracted DNA was first fragmented to yield -150 bp fragments with enzymatic digestion (RM0434, BGI Wuhan Biotechnology, Wuhan, China). For the construction of DNA library, fragmented DNA was further end-repaired, ligated to adapters and amplified using PCR with the PMseqTM high throughput gene detection kit for infectious pathogens (combined probe anchored polymerization sequencing method, RM0438, BGI-Shenzhen, Shenzhen, China), according to the manufacturer’s instructions. Based on the qualified double strand DNA library, single-stranded circular DNA library was then generated through DNA-denaturation and circularization. Then DNA nanoballs (DNBs) were formed by rolling circle amplification (RCA) using a universal kit for sequencing reaction (Combinatorial Probe-Anchor Synthesis, RM0170, BGI-Shenzhen, Shenzhen, China),. DNBs were qualified by Qubit^®^ ssDNA Assay Kit (Thermo Fisher Scientific) and were further sequenced by MGISEQ-2000 platform (MGI, China). High quality sequencing data were generated by removing low-quality, adapter contamination, and short (length < 35 base pairs [bp]) reads. The total number of sequencing reads was at least 20 million per library. Human host sequences mapped to the human reference genome (hg19) were then excluded by computational subtraction using Burrows–Wheeler alignment (Li and Durbin, 2009). The remaining reads were classified into four microbial genome databases (bacteria, fungi, viruses, and parasites) by simultaneously aligning to the Pathogens Metagenomics Database (PMDB). The classification reference databases were acquired from NCBI (ncbi.nlm.nih.gov/genomes/). RefSeq contains the whole genome sequence of 4,945 viruses, 6,350 bacteria, 1064 fungi related to human infections, and 234 parasites associated with human diseases.

### Criteria for a positive mNGS result

Given the lack of standards to interpret the reports of mNGS and the variety of different sequencing platforms, the following criteria were applied to define positive results: 1) possessing pulmonary pathogenicity reported in the literature; 2) bacteria: >30% relative abundance at the genus level for opportunistic pathogenic bacteria, ≥3 stringently mapped reads at the species level for high pathogenicity bacteria, ≥1 stringently mapped read for *Mycobacterium tuberculosis complex* and non-tuberculous mycobacteria (Langelier et al., 2018, Chen et al., 2021); 3) fungi (*Candida* excluded): ≥1 stringently mapped read at the species level or ≥10 stringently mapped reads for mold at the species level (Peng et al., 2021); 4) mycoplasma, chlamydia: ≥1 stringently mapped reads at the species level; 5) oral commensals, *Candida*, viruses: evaluated by a physician based on clinical manifestations and examination results (Peng et al., 2021).

### Statistical analysis

Continuous variables were reported as medians and interquartile ranges. Categorical variables were expressed as frequencies and percentages. The 95% confidence intervals of the proportions were calculated using Wilson’s method. McNemar’s test was used to compare the detection rates of CMTs and mNGS. Chi-square tests were used to evaluate the statistical difference between non-matched samples and odd radio. All statistical analyses were performed using SPSS25. All tests were two-tailed, and statistical significance was set at P < 0.05.

## Results

### Patient characteristics

The baseline characteristics of 502 patients recruited for this study were presented in **Table 1**. Two hundred and ninety-seven were males. The median age was 58.0 years. 104(20.7%) patients were classified into immunocompromised group. 93(18.5%) patients were classified into bronchiectasis group. 90 patients were classified into the other comorbidities group. 215(42.8%) patients were classified into simple pulmonary infection group. 306(61%) patients had already received therapy of antibiotics before hospitalization (the most common antibiotics were β-lactam and quinolones).

**Table 1:** Clinical characteristics of 502 patients.

Characteristics	Value
Age, years, median (Q1, Q3)	58.0(47.0, 68.0)
Gender, male, n (%)	297 (59.2%)
Antibiotic therapy before hospitalization	306 (61.0%)
**Comorbidities**	
Immunocompromised group, n (%)	104 (20.7%)
Autoimmune disease	30
Long-term therapy of steroids	25
Recent chemotherapy	21
Solid-organ transplantation	18
Hematological malignancy	7
Agranulocytosis	2
HIV	1
Bronchiectasis group, n (%)	93 (18.5%)
Bronchiectasis patients	91
Destructive pneumonophthisis	2
Other comorbidities group, n (%)	90 (17.9%)
Diabetes	37
Chronic obstructive pulmonary disease	30
Interstitial lung disease	6
Bronchial asthma	4
Cerebrovascular disease	6
Targeted therapy and surgical of lung cancer	4
Liver cirrhosis	3
Simple pulmonary infection group, n (%)	215 (42.8%)

### Infection types

According to a comprehensive analysis of medical records and the results of mNGS, 84.1% (422/502) of patients were diagnosed with pulmonary infection and 15.9% of (80/502) patients were considered non-infectious diseases, including malignancies, organizing pneumonia, and vasculitis. Among the infectious patients, 355 patients were detected the causative pathogens (including 49 patients with polymicrobial infection and 306 patients with monomicrobial infection), whereas 67 patients were not confirmed the causative pathogen. It is worth noting that polymicrobial infections most often occurred in the immunocompromised group (22.1%, [95%CI, 14.0%-30.2%]), followed by the bronchiectasis group (9.7%, [95%CI, 3.6%-15.8%]) and other comorbidities (13.3% [95%CI, 6.2%-20.5%]). However, only 2.3% (95%CI, 0.3%-4.4%) of patients in the simple pulmonary infection group had polymicrobial infection (**Table 2**), and the proportion of polymicrobial infections in the immunocompromised group was much higher than that in the simple pulmonary infection group (OR= 11.9 [95%CI, 4.4-32.4%]). Similar results were also observed in the bronchiectasis (OR= 4.5 [95%CI, 1.5-13.8%]) and other comorbidities group (OR= 6.5 [95%CI, 2.2-18.9%]).

**Table 2:** Infection types in different comorbidities.

	Pulmonary infection	Non-infection	Polymicrobial infection	Monomicrobial infections	Not confirmed pathogen
Total	422 (84.1%)	80 (15.9%)	49 (9.7%)	306 (61.0%)	67 (13.3%)
Immunocompromised group	98 (94.2%)	6 (5.8%)	23 (22.1%)	69 (66.3%)	6 (5.8%)
Bronchiectasis group	85 (91.4%)	8 (8.6%)	(9.7%)	60 (64.5%)	16 (17.2%)
Other comorbidities group	71 (78.8%)	19 (21.1%)	12 (13.3%)	45 (50.0%)	14 (15.6%)
Simple pulmonary infection group	168 (78.1%)	47 (21.9%)	5 (2.3%)	132 (61.4%)	31 (14.4%)

### Pathogen spectrum in different groups

**Figure 1:**
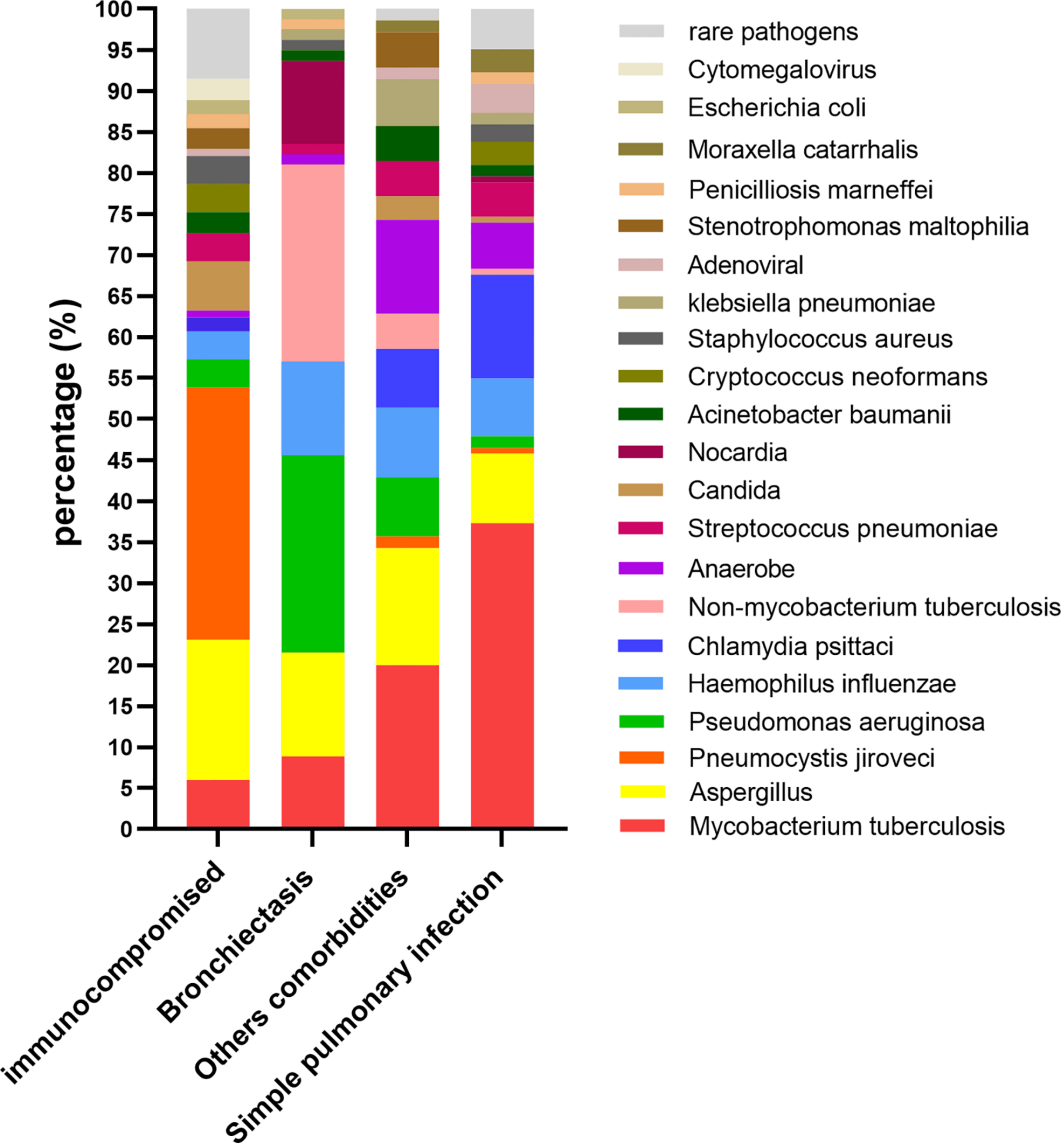
The percentage of different pathogens(y-axis) in patients with different comorbidities (x-axis).

The spectrum of pathogens varied among patients with different comorbidities. In the immunocompromised group, the most common pathogens were *Pseudomonas jirovecii* (31.0%) and *Aspergillus* (17.2%). In the bronchiectasis group, the most common pathogens were *Pseudomonas aeruginosa* (24.1%) and non*-Mycobacterium tuberculosis* (24.1%). The most common pathogens in the other comorbidities group were *Mycobacterium tuberculosis* (20.3%), *Aspergillus* (14.5%), and anaerobes (11.6%). The most common pathogens in the simple pulmonary infection group were *Mycobacterium tuberculosis* (37.3%) and *Chlamydia psittaci* (12.7%) (**Figure 1**).

### Comparison of mNGS and CMTs

**Figure 2:**
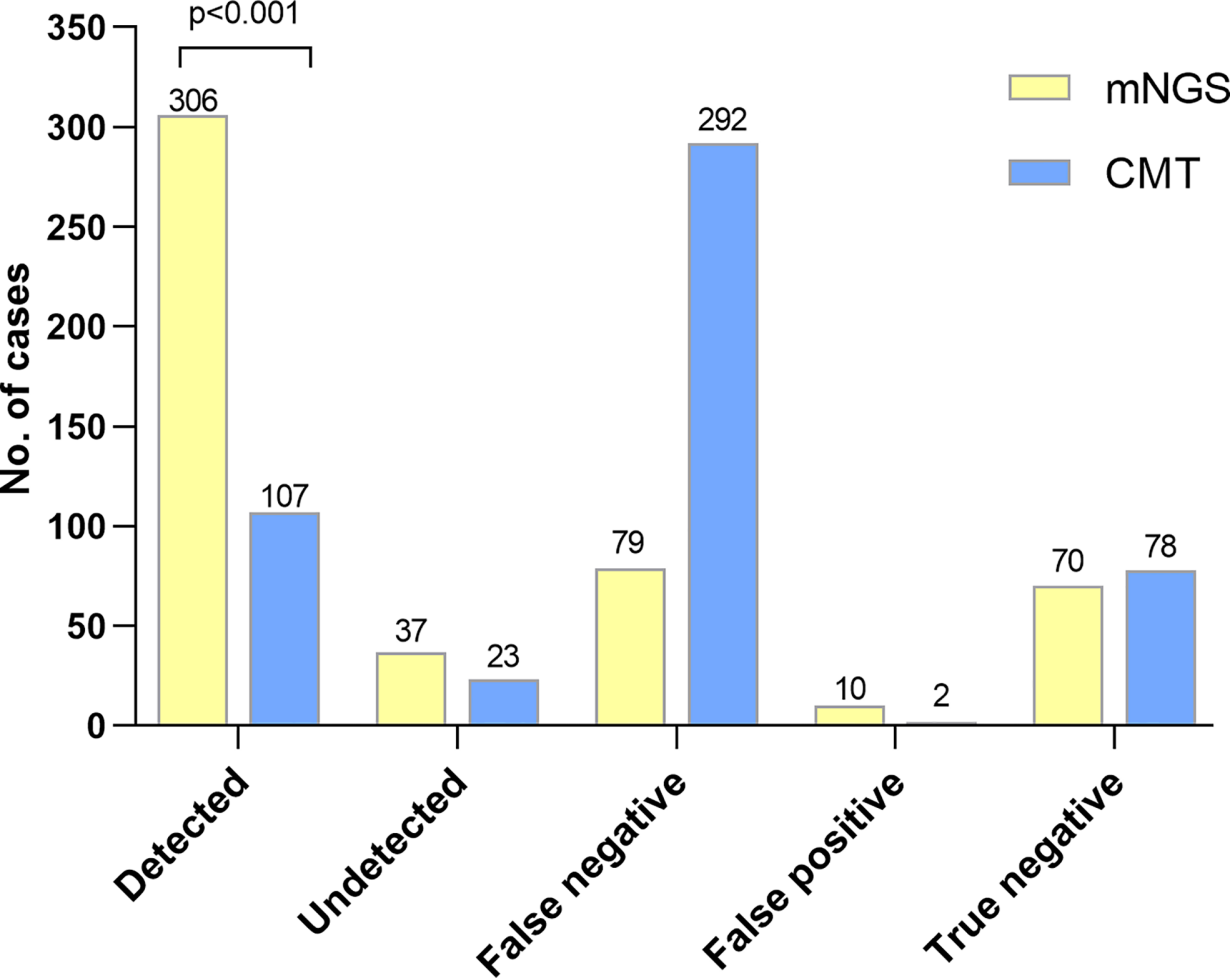
The diagnostic performance between mNGS and CMTs. Detection group: the causative pathogens were detected by mNGS or CMTs in infectious patients. Misdetection group: positive results by mNGS or CMTs were not corresponded with the reference standard in infectious patients. False negative: negative results but in non-infectious patients. False positive: positive results but in non-infectious patients. True negative: negative result by mNGS or CMTs in non-infectious patients. Patients were diagnosed with infectious or non-infectious diseases and the causative pathogens of infectious patients were determined according to comprehensive analysis of medical records. Causative pathogens detected by mNGS were more than by CMTs (p<0.001).

In the present study, 306 infectious patients obtained accurate microbiological diagnosis by mNGS. However, only 107 infectious patients were detected the causative pathogens by CMTs. In 37 infectious patients with positive results of mNGS, the detection results were inconsistent with the causative pathogens determined by a comprehensive analysis of medical records (**Figure 2**). Diagnostic accuracy was defined as proportion of correctly diagnosed patients (both infectious and non-infectious) among all patients. Sensitivity was defined as the proportion of infectious patients with correct diagnosis among the total number of infectious patients. The diagnostic accuracy of mNGS was much higher than that of CMTs (74.9% *vs.* 36.9%, p<0.001). The sensitivity of mNGS was also higher than that of CMTs (72.5% *vs.* 25.4%, p< 0.001). In addition, 70 of 149 patients with negative mNGS results were diagnosed with non-infectious diseases, and the negative predictive value (NPV) of mNGS was 47% (95%CI, 38.9%-55.1%) (**Table 3**).

**Table 3:** The diagnostic performance between mNGS and CMTs.

	Diagnostic accuracy	Sensitivity	NPV
	(95% CI)	(95% CI)	(95%CI)
mNGS	74.9(71.7-78.7)	72.5(68.2-76.8)	47.0(38.9-55.1)
CMTs	36.9(32.6-41.1)	25.4(21.2-29.5)	21.1(16.9-25.3)

**Figure 3:**
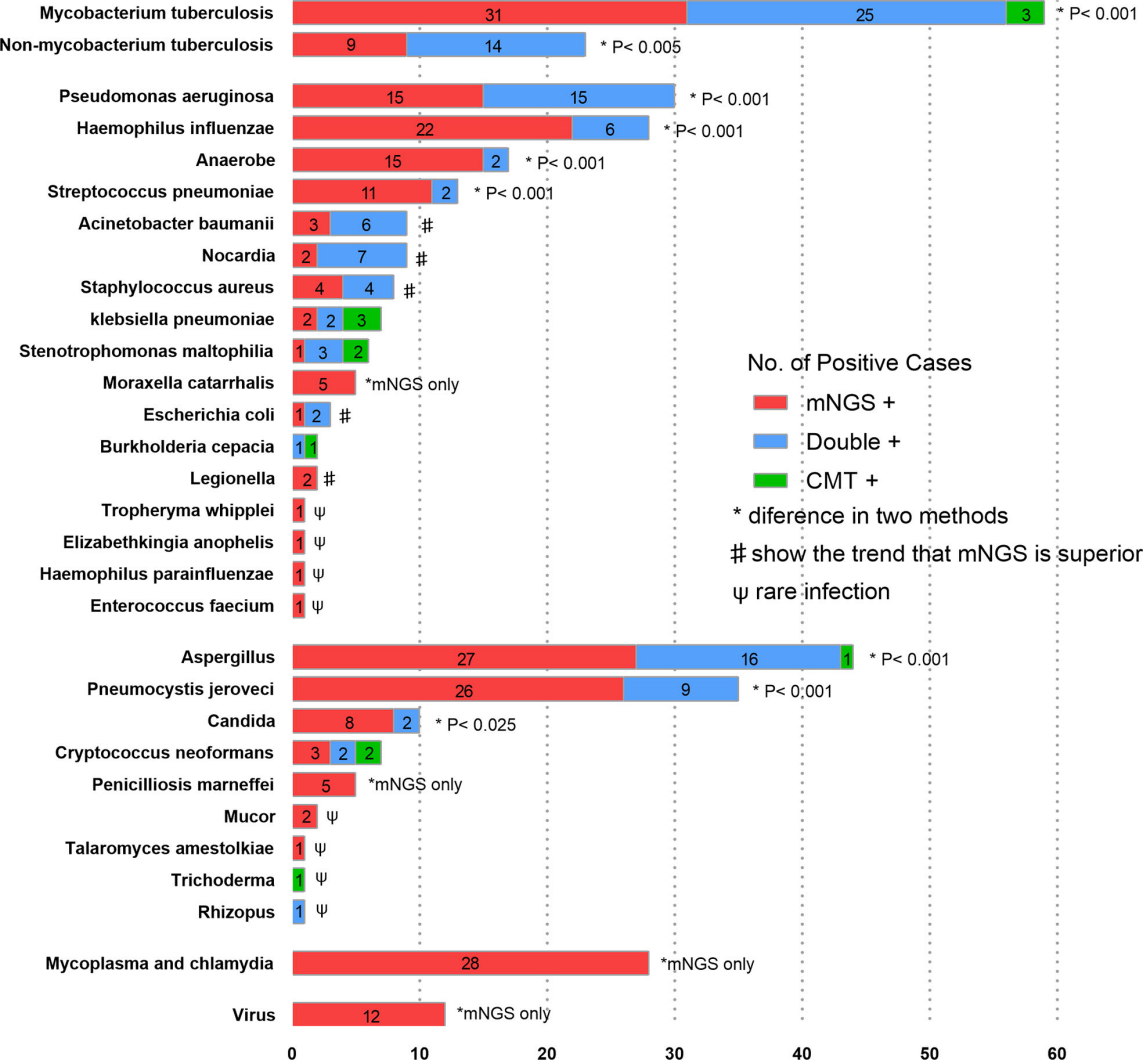
The overlap of positivity between mNGS and CMTs for different pathogens. Numbers in the histogram represents the corresponding cases of pathogens. In *Mycobacterium tuberculosis*, *Non-mycobacterium tuberculosis*, *Pseudomonas aeruginosa*, *Haemophilus influenzae*, *Anaerobe*, *Streptococcus pneumoniae*, *Aspergillus* and *Pneumocystis jirovecii*, the detection rates of mNGS were higher than those of CMTs. As for *Acinetobacter baumanii*, *Nocardia*, *Staphylococcus aureus* and *Escherichia coli*, there were no statistical differences, but mNGS tended to outperform CMTs.

Among 355 patients with microbiologically confirmed infection, 406 pathogens were detected (some of the patients were diagnosed with polymicrobial infection). 120(29.6%) pathogens were detected by mNGS and CMTs simultaneously, 239(58.9%) pathogens were detected by mNGS separately, 12(3.0%) pathogens were detected by CMTs separately, and 35(8.6%) pathogens were confirmed by other methods. The pathogen detection rate of mNGS was much higher than that of CMTs (88.4% *vs.* 32.5%, p< 0.001). As shown in **Figure 3**, in most pathogens, mNGS also showed better diagnostic performance than CMTs, especially in *Mycoplasma, Chlamydia* and *viruses* which were exclusively detected by mNGS. For *Acinetobacter baumannii*, *Nocardia*, *Staphylococcus aureus*, *Escherichia coli* and *Legionella*, there was no statistical difference between the two methods because of the small sample size. However, these pathogens also showed at trend that mNGS was superior to CMTs.

### The PPV of pathogens in mNGS reports

**Figure 4:**
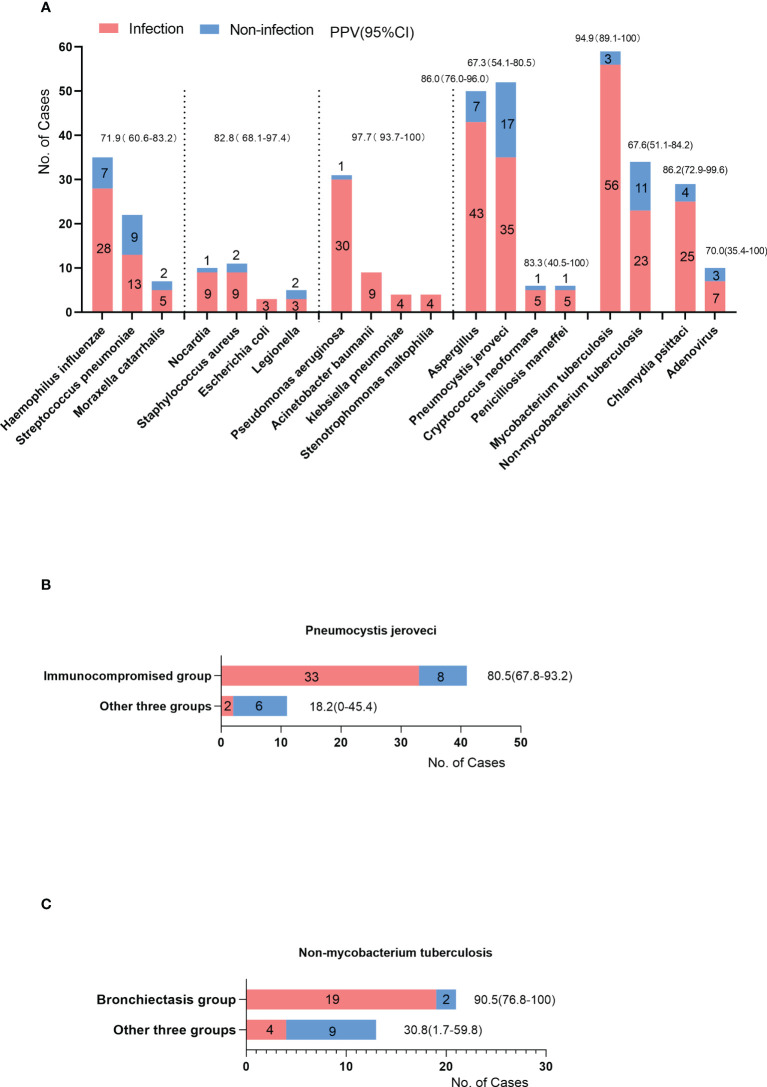
**(A)** The cases of positive results of mNGS (y-axis) in different pathogens (x-axis). Some were the causative pathogens (infection cases), others were considered environmental contaminants or colonized microbes (non-infection cases). The PPV was the proportion of infection cases among the cases of positive results. The PPVs and 95%CI were listed on the top of the bars. **(B)** The PPV of *Pneumocystis jirovecii* in immunocompromised group compared with other three groups. **(C)** The PPV of *Non-mycobacterium tuberculosis* in bronchiectasis group compared with other three groups.

To evaluate the reliability of positive mNGS results, the PPV of mNGS was calculated for different pathogens. Bacteria were classified into different groups according to their pathogenicity and the possibility of colonizing the respiratory tract. *Haemophilus influenzae*, *Streptococcus pneumoniae*, and *Moraxella catarrhalis* are the most common pathogens causing community-acquired pneumonia, which can also colonize the nasopharynx (Teo et al., 2015). The summarized PPV was 71.9% (95%CI, 60.6-83.2%). *Nocardia, Staphylococcus aureus, Escherichia coli*, and *Legionella* are highly pathogenic to the respiratory tract. Their summarized PPV was 82.8% (95%CI, 68.1-97.4%). Unlike previous bacteria, *Pseudomonas aeruginosa, Acinetobacter baumannii, Klebsiella pneumoniae* and *Stenotrophomonas maltophilia* can colonize the lower respiratory tract. Their summarized PPV was 97.7% (95%CI, 93.7-100%). The PPVs of *Aspergillus* and *Pneumocystis jirovecii* were 86.0% and 67.3%, respectively. Among mycobacteria, the PPVs of *Mycobacterium tuberculosis* and *Non-mycobacterium tuberculosis* were 94.9% and 67.6%, respectively (**Figure 4A**). Notably, the immunocompromised group had a higher PPV of *Pneumocystis jirovecii* than the other three groups (80.5% *vs.* 12.8% p=0.006) (**Figure 4B**). Similarly, the PPV of non*-Mycobacterium tuberculosis* in the bronchiectasis group was higher than that in the other three groups (90.5% *vs.* 30.8%, p=0.001) (**Figure 4C**).

## Discussion

In this study, 502 patients with suspected pulmonary infections were retrospectively analyzed. According to a comprehensive analysis of medical records, 422 (84.1%) patients were diagnosed with pulmonary infection and 80 (15.9%) were considered to have non-infectious diseases. Based on comorbidities, patients were classified into four groups. The spectrum of pathogens and proportion of polymicrobial infections varied among the different groups. Moreover, we systematically compared the diagnostic performance of mNGS of BALF and CMTs; the former was significantly superior to the latter. In addition, it can also be demonstrated that the PPVs of mNGS varied in different types of pathogens, which should be considered when interpreting the results of mNGS.

The diagnostic accuracy and sensitivity of mNGS were 74.9% (95%CI, 71.7-78.7%) and 72.5% (95%CI, 68.2-76.8%), respectively, which were much higher than those of CMTs. In a study of 235 patients with suspected pneumonia, the sensitivity of BALF mNGS reached up to 73.33% (Chen et al., 2021). In another study of 132 patients, compared to conventional testing, mNGS suggested potentially missed diagnoses in 22 patients involving 48 additional pathogenic microorganisms (Zhan et al., 2021). This study also demonstrated that the detection rate of mNGS was higher than that of CMTs in most of the pathogens. In a retrospective study of 72 patients, the detection rate of bacteria by mNGS was also higher than conventional methods. As for fungi, the detection rate of mNGS is reported comparable to conventional tests because of the introduction of Galactomannan antigen and (1,3)-β-D-glucans (Fang et al., 2020). However, these two methods are not regarded as etiologically confirmed tests because they cannot indicate the specific species of pathogens. When the two methods were excluded, the detection rate of mNGS was higher than that of conventional tests for fungi (Miao et al., 2018).

Unlike previous studies (Chen et al., 2021; Peng et al., 2021), we did not observe a high NPV value (47.0%, 95%CI [38.9-55.1%]). For example, in a retrospective study, the NPV for different pathogens varied from 73.5% to 100% (Peng et al., 2021). Another study demonstrated that the NPV of mNGS of BALF was 85.88% (95%CI,76.25–92.18%) (Chen et al., 2021). There are explanations for these conflicting results. *Mycobacterium tuberculosis* was the most common pathogens in this study. The detection of *Mycobacterium tuberculosis* requires cell wall disruption to release nucleic acid (Simner et al., 2018). A low biomass in DNA extraction can also decrease the NPV. Moreover, rare pathogens (like *Kingella, Tropheryma whipplei*) and viruses (such as *Human herpes virus* and *Torque teno virus*) are usually not regarded as causative pathogens unless they are considered significant by the managing clinicians. To the best of our knowledge, this research is the largest sample size investigation to evaluate the diagnostic performance of mNGS for BALF in patients with pneumonia.

Immunocompromised patients have a more complex microbial etiology, with a higher detection rate of *Pneumocystis jirovecii* and *Aspergillus*. *Pseudomonas aeruginosa* and non*-Mycobacterium tuberculosis* were the dominant pathogens in patients with bronchiectasis. *Pseudomonas aeruginosa* was the most common pathogen isolated from the respiratory tract specimens of patients with bronchiectasis. Non*-Mycobacterium tuberculosis* has been increasingly detected globally (To et al., 2020). In this study, 40.0% (14/35) of the patients with positive acid-fast stains were diagnosed with non*-Mycobacterium tuberculosis* infection by mNGS. Since not all patients with positive acid-fast staining had *Mycobacterium tuberculosis* infection, and the culture of *Mycobacterium* takes one to three months, mNGS might be an efficient method to distinguish non*-Mycobacterium tuberculosis* infection (Shi et al., 2020). *Mycobacterium tuberculosis* was the most common pathogen in the patients without these comorbidities. The high detection rate of this bacterium is reasonable. First, there is a high prevalence of *Mycobacterium tuberculosis* infection in China (Organization, W.H. 2021). Moreover, most of the patients enrolled in our study received empirical anti-infective treatment. Interestingly, a significant number of patients in the simple pulmonary infection group were diagnosed with *Chlamydia psittaci* infection, which may be associated with universal poultry production in Jiangxi, China (Li et al., 2021).

Another strength of our study is that we strived to evaluate the PPVs of mNGS for different types of pathogens, which can help clinicians interpret the reports of mNGS. The summarized PPV of *Haemophilus influenzae*, *Streptococcus pneumoniae*, and *Moraxella catarrhalis* were 71.9% (95%CI, 60.6-83.2%). As the most common colonizing bacteria in the nasopharynx (Teo et al., 2015), these pathogens can be carried into the BALF during bronchoscopy. Thus, attention should be paid to distinguish between colonization and infection based on the positive results of these pathogens. The summarized PPV of *Pseudomonas aeruginosa, Acinetobacter baumannii, Klebsiella pneumoniae*, and *Stenotrophomonas maltophilia* were 97% (95%CI, 93.7-100%). This high value is mainly due to a stricter positive criterion in reports of mNGS; these bacteria were not considered a positive result of mNGS if their relative abundance at the genus level was less than 30%. A similar high PPV of these pathogens was observed in Peng et al.’s study (Peng et al., 2021). It should be noted that the PPV of *Pneumocystis jirovecii* in the immunocompromised group was much higher than that in the other three groups (80.5%–18.2%, p=0.006). The positive result for *Pneumocystis* *jirovecii* in immunocompromised patients is more likely to be the causative pathogen. In contrast, the positive result of *Pneumocystis jirovecii* in immunocompetent patients is more likely to be considered environmental contaminants. Similar features can be observed in non*-Mycobacterium tuberculosis*. Therefore, the commodities and types of pathogens should be considered when interpreting mNGS reports.

The potential for pathogen detection using mNGS has been confirmed in many studies and clinical contexts. However, there are still limitations to the application of mNGS in clinical settings. Multiple evidence suggested that the respiratory tract is not a sterile environment. Commensal microbes commonly exist in the respiratory tract of healthy individuals, indicating that microbes with many reads in mNGS reports are not always the causative pathogen (Dickson et al., 2017). In addition, the existence of environmental contaminants, opportunistic pathogens, and mismatch of DNA fragments also makes it difficult to interpret the reports of mNGS. Therefore, a reasonable and accurate interpretation is an important bottleneck (Han et al., 2019). The cost is another concern for the widespread use of mNGS. In China, the current cost is approximately $600 per sample, which is much higher than that of conventional tests (Miao, Ma et al., 2018, Fang, Mei et al., 2020). Currently, a vast proportion of the reads (>90%) sequenced by mNGS are host-derived, which means most of money is spent on these invalid sequences. Reducing the host reads prior to sequencing using host depletion methods can effectively reduce the total cost (Marotz et al., 2018). Increasing the number of test samples per run can also reduce the cost per sample, but it comes at the expense of turnaround time (Rutanga et al., 2018). In addition, encouraging more high-quality sequencing platforms to participate in the market can indirectly reduce costs.

This study has several limitations. First, it is a retrospective study of two center, which may lead to potential selection bias. Second, we failed to conduct cultures for *Mycobacterium tuberculosis* and non*-Mycobacterium tuberculosis*. Third, the patients classified into other comorbidity groups had different comorbidities (e.g. diabetes, chronic obstructive pulmonary disease, interstitial lung disease, cerebrovascular disease, lung cancer treated with targeted therapy or surgical, liver cirrhosis), which may be associated with the heterogeneity of the spectrum of pathogens. Forth, most patients enrolled in the present study have received anti-infective therapy before hospitalization, which might result in the underestimation of culture sensitivity and higher chance of infections caused by opportunistic pathogens. Finally, the interpretation of the mNGS results depends on the clinician’s subjective judgment, which may lead to bias.

Overall, the application of mNGS to BALF can improve pathogen detection in patients with pulmonary infections. However, the interpretation of reports and the cost of mNGS remain concerns. This study demonstrated that the commodities and types of pathogens should be considered when interpreting mNGS reports. Further investigations are still needed for the extension of mNGS.

## Data availability statement

The datasets presented in this study can be found in online repositories. The names of the repository/repositories and accession number(s) can be found below: https://db.cngb.org/search/project/CNP0002658/.

## Ethics statement

The studies involving human participants were reviewed and approved by Ethics Committee of Second Affiliated Hospital of Nanchang University (No. 2019-013) Jiangxi Provincial People’s Hospital (No.2021-076). The patients/participants provided their written informed consent to participate in this study.

## Author contributions

DW and WW analyzed the data, drafted the first version of the manuscript, and submitted it to the publication; QX and HW collected data and helped to analyze data; JL, ZZ, and CO helped to collect data; QY was responsible for the entire project, designed the experiment, and revised the draft of the manuscript. All authors contributed to the article and approved the submitted version.

## Funding

This work was supported by the National Natural Science Foundation of China (Grant no. 81860011).

## Acknowledgments

We would like to acknowledge the patients for cooperating with our investigation.

## Conflict of interest

The authors declare that the research was conducted in the absence of any commercial or financial relationships that could be construed as a potential conflict of interest.

## Publisher’s note

All claims expressed in this article are solely those of the authors and do not necessarily represent those of their affiliated organizations, or those of the publisher, the editors and the reviewers. Any product that may be evaluated in this article, or claim that may be made by its manufacturer, is not guaranteed or endorsed by the publisher.
